# Assessing plate reconstruction models using plate driving force consistency tests

**DOI:** 10.1038/s41598-023-37117-w

**Published:** 2023-06-23

**Authors:** Edward J. Clennett, Adam F. Holt, Michael G. Tetley, Thorsten W. Becker, Claudio Faccenna

**Affiliations:** 1 Institute for Geophysics, Jackson School of Geosciences, The University of Texas at Austin, Austin, USA; 2 Department of Geological Sciences, Jackson School of Geosciences, The University of Texas at Austin, Austin, USA; 3 Rosenstiel School of Marine, Atmospheric, and Earth Science, University of Miami, Miami, USA; 4 Oden Institute for Computational Engineering and Sciences, The University of Texas at Austin, Austin, USA; 5 Dipartimento Scienze, Università Roma Tre, Rome, Italy; 6 GFZ Helmholtz Centre Potsdam, German Research Centre for Geosciences, Potsdam, Germany

**Keywords:** Tectonics, Geodynamics, Geophysics

## Abstract

Plate reconstruction models are constructed to fit constraints such as magnetic anomalies, fracture zones, paleomagnetic poles, geological observations and seismic tomography. However, these models do not consider the physical equations of plate driving forces when reconstructing plate motion. This can potentially result in geodynamically-implausible plate motions, which has implications for a range of work based on plate reconstruction models. We present a new algorithm that calculates time-dependent slab pull, ridge push (GPE force) and mantle drag resistance for any topologically closed reconstruction, and evaluates the residuals—or missing components—required for torques to balance given our assumed plate driving force relationships. In all analyzed models, residual torques for the present-day are three orders of magnitude smaller than the typical driving torques for oceanic plates, but can be of the same order of magnitude back in time—particularly from 90 to 50 Ma. Using the Pacific plate as an example, we show how our algorithm can be used to identify areas and times with high residual torques, where either plate reconstructions have a high degree of geodynamic implausibility or our understanding of the underlying geodynamic forces is incomplete. We suggest strategies for plate model improvements and also identify times when other forces such as active mantle flow were likely important contributors. Our algorithm is intended as a tool to help assess and improve plate reconstruction models based on a transparent and expandable set of a priori dynamic constraints.

## Introduction

Plate reconstruction models are widely used as both inputs and constraints for studies in a range of different scientific fields, including climate and ocean circulation modelling^1,2^, natural resource prospecting^3–5^, and geodynamic modelling^6,7^. These kinematic models use magnetic anomalies, fracture zones and hotspot tracks on surviving seafloor, combined with continental paleomagnetic data and geological evidence, to describe the motion of the Earth’s surface through deep time^8–10^. However, in contrast to geodynamic forward models, plate reconstructions are designed to be geometrically self-consistent, i.e. motions in these models are prescribed to match often incomplete constraints on the kinematics, rather than generated self-consistently by solving the conservation equations guiding the dynamics. This can lead to motions in plate reconstructions that are inconsistent with geodynamic considerations, particularly for poorly constrained components of the model such as in reconstructed oceanic lithosphere for times before ~ 100 Ma^11^.

Plate reconstructions can be evaluated in several ways, including using surface and mantle constraints, and on different scales from regional to global. Seismic tomography presents a record of convection, and either circulation models or kinematic approaches can be used to test the consistency of a given set of plate motions with these observations of Earth’s interior^12–14^. For example, Shephard et al.^15^ tested different absolute reference frames against mantle structure by comparing the predicted mantle structure with seismic tomography. Alternatively, Williams et al.^16^ took a statistical approach, comparing metrics for plate speeds, trench motions, and net lithospheric rotation for different absolute plate motion models; they identified the ‘optimum’ reference frames as those that have the lowest trench velocities and net rotations, consistent with present-day constraints^17–19^.

Building on these statistical tests, Tetley et al.^20^ and subsequently Müller et al.^10,21^ developed an iterative approach to optimize reference frame consistency with both observational and geodynamic constraints. Tetley et al.^20^ showed that observational constraints from hotspot tracks can be matched whilst satisfying a priori assumptions of minimizing rates of trench migration, net lithospheric rotation, and absolute plate velocities. Here, we seek to advance such work toward geodynamically constrained plate reconstructions by providing a tool to calculate the time-dependent plate driving force balance for plate reconstructions. This tool can be used to explore driving force consistency and we discuss results for a number of modern plate reconstructions.

**Figure 1:**
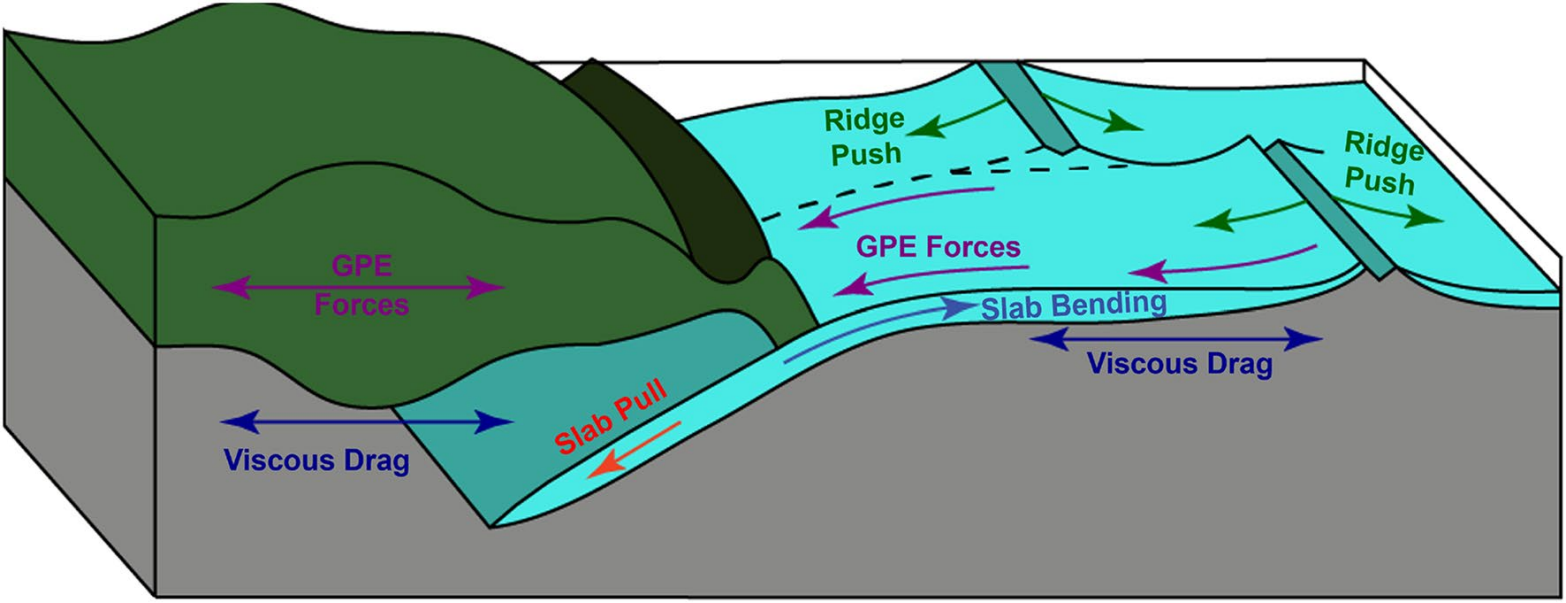
Plate driving forces calculated in this study. Figure adapted from Forsyth and Uyeda^22^.

Tectonic plates are primarily driven by: the negative buoyancy of subducting oceanic lithosphere, known as “slab pull”; forces resulting from lateral gradients in gravitational potential energy (GPE), such as within oceanic lithosphere where it is somewhat misleadingly called “ridge push”; and distributed tractions due to mantle flow (Fig. 1). Slab pull is generally accepted to be the dominant driving force for the present-day plate system, as subducting oceanic plates such as the Pacific, Nazca and Cocos plates have significantly higher velocities than overriding plates^22^. This is further supported by theoretical calculations, which show slab pull to be an order of magnitude stronger than ridge push^23^, and torque balances, where slab pull alone can explain 90% of the direction and magnitude of present-day plate motion^22,24–26^. However, for certain plates, particularly those that are not attached to large masses of subducting slabs, there can be multiple candidates for primary drivers of plate motion, including GPE forces and active mantle flow. For example, recent motion of the Indian plate has been attributed to slab pull at the Sunda subduction zone^27,28^, GPE forces^29,30^, mantle tractions exerted by a whole mantle convection cell^31^, and a potential plume push force^32,33^.

Although relatively well understood at present-day, the influence of slab pull and other forces is less well constrained the further back in time. Previous work investigating this on a global scale has utilized mantle circulation modelling^24,34,35^, where plates are driven primarily by slab pull and mantle flow arising from dense subducted slabs in the upper and lower mantle. These studies can reproduce Cenozoic plate motions to within correlations of 70–90%, but do not reconcile observed rapid plate reorganizations and motion changes, such as the Hawaiian-Emperor Bend (HEB) in the Pacific plate.

Previous time-dependent plate driving force studies have focused specifically on the Pacific plate to investigate this problem. Faccenna et al.^36^ concluded that slab pull forces alone can explain the direction of motion of the Pacific plate during the Cenozoic, with the onset of subduction at the Izu-Bonin-Mariana system causing the change in plate motion recorded in the HEB. Furthermore, Hu et al.^37^ tested different Pacific subduction zone configurations using sophisticated global mantle flow models, concluding that plate motions and plate driving forces.are best reconciled when including the suggested Kronotsky intra-oceanic subduction in the North Pacific^38–42^. However, considering slab pull alone, the magnitude of plate velocity over time is poorly matched; in particular, there are times of significant motions without identified slab pull. Identification of this mismatch is consistent with other studies which suggest that forces associated with mantle flow are major drivers of Pacific plate motions through time. For example, slab suction—a mantle flow force driven by upper and lower mantle slabs—has been hypothesized to account for 60% of the early Cenozoic driving force^35^; active pressure-driven flow in the asthenosphere could have driven at least 50% of the Pacific plate motion over the past 15 Myr^43^; and a deep mantle buoyancy associated with the East Pacific Rise could account for a major component of the Pacific plate motion since 80 Ma^44^.

Currently, there are no assessments of different published plate reconstructions that are based on plate driving forces, with most previous studies of driving forces focusing on a single reconstruction model^24,35^ or plate^36,37,43,44^. However, the development of the plate modelling software, *GPlates*^45^, in recent years has led to a sharp increase in the number of plate reconstruction models that are easily comparable quantitatively. We developed an algorithm to calculate plate driving forces through time for any topologically closed plate reconstruction model (global or regional), with the option for users to easily change parameters to suit their needs. This will allow users to identify parts of reconstruction models that might be inconsistent with geodynamic drivers and need studying in more detail, or estimate how the driving forces acting on a specific plate have changed over time.

## Plate driving force calculations

**Figure 2:**
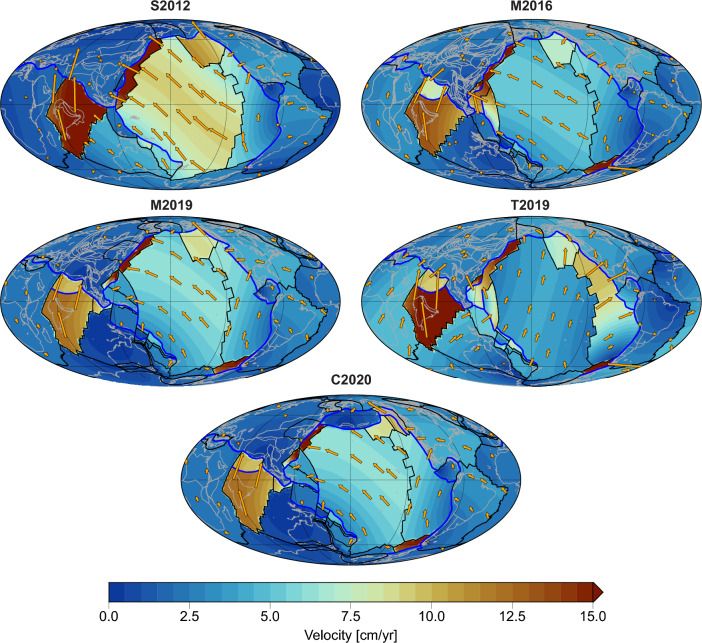
Comparison of the five plate reconstruction models analyzed in this study at 60 Ma. Bold blue lines are subduction zones, black lines are ridge-transform boundaries, and continents are shown in light grey. The colourmap shows the plate velocity, highlighting the differences in absolute and relative plate motion between models.

To extract plate geometries, plate boundary type, and motions from individual plate models, we use *pyGPlates*^45^, the Python application programming interface (API) of *GPlates*. We analyzed five global plate reconstructions that are provided in *GPlates* format, which are based on a variety of constraints including hotspot tracks, paleomagnetic poles, seismic tomography and considerations of net rotation and trench migration. Different weighting of these constraints can lead to considerable variations in plate geometries and velocities at a given time, which is highlighted in Fig. 2.

Three of these plate models—Seton et al.^9^ (hereafter S2012), Muller et al.^46^ (M2016) and Torsvik et al.^47^ (T2019)—incorporate a hybrid hotspot/paleomagnetic reference frame, where relative plate motions at recent times are anchored to a global moving hotspot model and plate motions for older times are constrained using paleomagnetic data. The three plate reconstructions all use different hotspot frames and T2019 also uses a different paleomagnetic frame to S2012 and M2016, leading to differences in absolute plate motions across the models. Relative plate motions also vary between models, due to differing interpretations of geological.and geophysical observations. Müller et al.^10^ (M2019) used a geodynamically-optimized mantle reference frame^20^, which minimizes net rotation and trench migration whilst still fitting hotspot tracks. In addition, this model includes deforming regions, which allows for changes in geometry and crustal thickness. The plate reconstruction of Clennett et al.^42^ (C2020) is a regional refinement of western North America and the northern and eastern Pacific basin where subduction zones and plate motions are constrained using seismic tomography, paleomagnetism and geological evidence. C2020 builds on M2019, and so these two models are identical for plates away from the eastern Pacific region.

We generated seafloor age grids using the method of Williams et al.^48^ and sampled these grids to obtain the seafloor ages. At 1 Myr intervals, we then used the plate boundary lengths, plate areas and seafloor ages, together with the parameters listed in Supplementary Table S1 in our calculations of slab pull, GPE force and mantle drag resistance. We also calculated the residual torque—the missing torque required for all torques to sum to zero—and converted this back to a total force acting at the centroid. This methodology is detailed in the following sections.

### Slab pull

Slab pull is the force due to the negative buoyancy of a subducting slab as it sinks through the mantle, which can be given by:

1 $$\overset\rightharpoonup{F}_{SP} = C\Delta \rho glh_{lith} \widehat{n}$$

Here, Δ*ρ* is the density contrast between the slab and the mantle, given by *ρ*_*m*_*α*Δ*T* where *ρ*_*m*_ is the mantle density, *α* is the coefficient of thermal expansivity, and Δ*T* is difference in temperature between the slab and the mantle; *g* is the acceleration due to gravity; *l* is the length of the slab; *h*_*lith*_ is the thickness of the slab, which varies as a function of plate age, *A*; $\widehat{n}$ is a horizontal unit vector normal to the trench and *C* is a constant that accounts for the reduction in the net force due to conductive heating or resistive stresses such as the slab encountering a higher viscosity lower mantle^23,49^.

Additional, resistive stresses may occur due to bending of the plate as it subducts^49^, and we implemented two versions of this bending force assuming either a viscous and visco-plastic rheology^50^. However, the inclusion of either form of plate bending only led to a < 10% difference in the total driving force on average, so we decided to simplify the resistive stresses to just a constant $C$, which we varied between 0.05 and 1.

In addition, we tested different methods of determining the length of the subducted slab. Taking the initiation age of each subduction zone from *GPlates*, we calculated an approximate slab length by multiplying the convergence velocity with the time passed since the start time. This did not improve the fit with plate motions compared with using a constant slab length—the residual force was either the same or slightly higher; thus, following Faccenna et al.^36^, we simplified the slab length to a constant 700 km.

We also implemented two ways to calculate the thickness of the oceanic lithosphere, *h*_*lith*_: the half-space cooling model and the modified plate model. The half-space cooling model uses a value of $2.32\sqrt{\kappa A}$, where the constant arises from the definition of the thermal boundary layer as the region where the temperature is less than 90% of the asthenospheric temperature; the modified plate model deviates from half-space cooling for oceanic lithosphere older than 81 Ma according to the following equation for water depth^51,52^:

2 $$d_{w} = \left\{ {\begin{array}{*{20}c} {2600\;{\text{m}} + 345\;{\text{m}}\;\sqrt A } & {\quad {\text{for}}\;\;A < 81\;{\text{Ma}}} \\ {6586\;{\text{m}} - 3200\;{\text{m}}\;\exp \left( {\frac{ - A}{{62.8}}} \right)} & {\quad {\text{for}}\;\;A > 81\;{\text{Ma}}} \\ \end{array} } \right.$$

Lithospheric thickness is then determined from isostasy, using the densities and compensation depth in Supplementary Table S1 and the water depth calculated in Eq. (2). Both equations are implemented into the code; as the residuals for the plate model were 3.5% lower than those for half-space cooling over the past 20 Myr (Supplementary Fig. S1), we use Eq. (2) when calculating plate driving forces in our analysis.

### GPE force (ridge push)

Ridge push is the lithospheric thickening force due to pressure differences between oceanic seafloor of varying age and thickness, and hence can be thought of as a gravitational sliding force. For a 1D half-space cooling model, this yields a force per unit length acting perpendicular to the ridge, which depends on the density contrast, $\Delta \rho (={\rho }_{m}\alpha\Delta T)$, gravity, $g$, thermal diffusivity, $\kappa$, and the age of the seafloor, *A*^23^:

3 $$\overset\rightharpoonup{F} _{RP} = g\rho_{m} \alpha \Delta T\left[ {1 + \frac{2}{\pi }\frac{\Delta \rho }{{(\rho_{m} - \rho_{w} )}}} \right]\kappa A\;\widehat{n}$$

However, as the seafloor age gradient is not always perpendicular to the spreading ridge, it is more realistic to model lithospheric thickening as tractions acting over the entire area of the plate. Therefore, we use the force per unit area that arises due to gradients in GPE $\nabla U$^53,54^.

4 $$\overset\rightharpoonup{\sigma }_{GPE}= - \frac{L_{0}}{L}\nabla U$$

Here, *L* is the isostatic compensation depth, *L*_0_ is the lithospheric shell thickness, and the GPE field is calculated by integrating over the height, *h*, of isostatically balanced columns^54^:

5 $${\Delta U = g\int_{h}^{L} {z \Delta \rho (z) dz} }$$

In oceanic regions, we calculate the thickness of the lithosphere using both half-space cooling and the modified plate model (Eq. 2). As in the slab pull calculation, we used the modified plate model of lithospheric thickness for our torque balance, although both methods are included in our code. In addition, we calculated the total GPE force by including variations in continental GPE. At the present day, continental GPE was calculated from a crustal thickness compilation^55^. However, in the past, crustal thickness is poorly constrained; M2019 includes several deforming regions in the plate model, but these are spatially and temporally limited, resulting in uncertainty in the continental GPE force through time.

**Figure 3:**
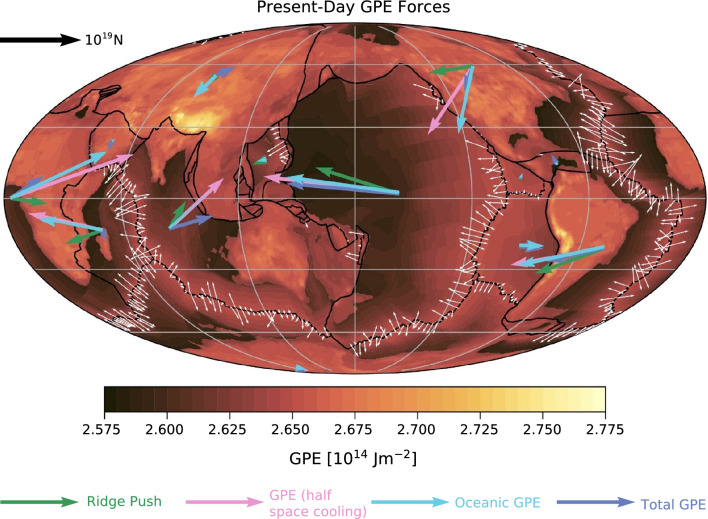
Comparison of the different ways of calculating the lithospheric thickening—or ridge push—forces acting on the Earth’s plates. The small white vectors show the 2D ridge push forces at the mid ocean ridge calculated from Eq. (3), which is integrated over the total ridge length to yield the green ridge push vector. The other vectors are all derived from Eqs. (4) and (5), with the pink vector derived from a half-space cooling model for oceanic lithospheric thickness, the cyan vector using a GPE field based on the plate model (Eq. 2), and the blue vector also including continental GPE as well as oceanic. This is for the M2016 model, but only for the 13 major plates, including a combined Indo-Australian plate.

Figure 3 shows the present-day GPE field calculated using Eq. (5) for the whole Earth. The total force acting at the plate centroid is also shown, highlighting the difference between 1D ridge push (Eq. 3) and 2D GPE (Eq. 4), as well the effect of different seafloor age profiles or variation in continental crustal thickness^25^. While the gradient-based approach is more physically realistic, it relies on reconstructed seafloor age grids for the past which introduce their own uncertainties. We discuss our preferred choice of GPE force in the optimization section (section “Present-day optimization”).

### Mantle flow

Lastly, tractions on the base of the lithosphere due to mantle flow contribute to the torque balance. While we can compute mantle circulation driven by plate motions themselves^56,57^ and other mantle density anomalies^58,59^, retrodicting mantle density structure back in time, and hence isolating active mantle flow contributions, is more involved and subject to uncertainties^60,61^. Therefore, in this study, we neglect active mantle flow by assuming mantle drag to be only a resisting force opposite to plate motions. The utility of this approach, relative to one which includes global convection modelling, is to simplify the calculation (i.e. reduction in parameters) and reduce computational cost. We use a simple Couette flow model for mantle drag, where tractions arise due to viscous shearing of the asthenosphere by plate motion. The resulting tractions are dependent on the thickness, *H*_*A*_, and viscosity, $\eta_{A}$, of the asthenosphere, and on the direction of plate motion, $\mathop{v}\limits^{\rightharpoonup} (\mathop{r}\limits^{\rightharpoonup} )$, as follows:

6 $$\overset\rightharpoonup{\sigma}_{MD} = \frac{\eta_{A}}{H_{A}}\overset\rightharpoonup{v} (\overset\rightharpoonup{r} )$$

In this study, we varied the drag coefficient, $D = \eta_{A} /H_{A}$, to find the optimum value to balance the driving force.

### Torque balance

To determine the torque acting on each plate, we take the cross product between the position vector, $r$, and the forces and tractions at each point. We then integrate the slab pull torque over the length of each subduction zone, and integrate the GPE and mantle drag torques over the plate area^25,26^, which gives the total torque acting on the plate.

7 $$\overset\rightharpoonup{\tau}_{total} = \int_{A}^{ } \overset\rightharpoonup{r} \times \overset\rightharpoonup{\sigma}_{GPE} \,dA + \int_{A}^{ } \overset\rightharpoonup{r} \times \overset\rightharpoonup{\sigma} _{MD} \,dA + \mathop \sum \limits_{ }^{N_{SZ}} \mathop \int \nolimits_{{L_{SZ} }}^{ } \mathop{r}\limits^{\rightharpoonup} \times \overset\rightharpoonup{F}_{SP} \,dl$$

In equilibrium, the sum of all the torques acting on the plate must equal zero^62^. We therefore calculate the residual torque—the missing forces required for plate motion to be in dynamic equilibrium,

8 $$\overset\rightharpoonup{\tau}_{residual} = - \overset\rightharpoonup{\tau}_{total} ,$$

as a quantitative indicator of how well the motion of a certain plate fits with this description of plate driving forces.

## Present-day optimization

To do this, we varied both the slab pull reduction factor and the drag coefficient—two parameters that are linearly related to each other for each plate, with a higher mantle drag required to balance a stronger slab pull force. In this optimization, GPE is held constant, which helps to set the scale for the optimum slab pull and mantle drag coefficients. We then selected the two values that minimized the area-weighted mean residual torque (Eqs. 7 and 8) for the major oceanic plates (Pacific, Nazca and Cocos); the residual torque for each plate was first normalized by the driving torque magnitude (i.e. slab pull plus GPE tractions) to ensure that there was no bias to smaller slab pull reduction factors, before being plotted on a log scale.

**Figure 4:**
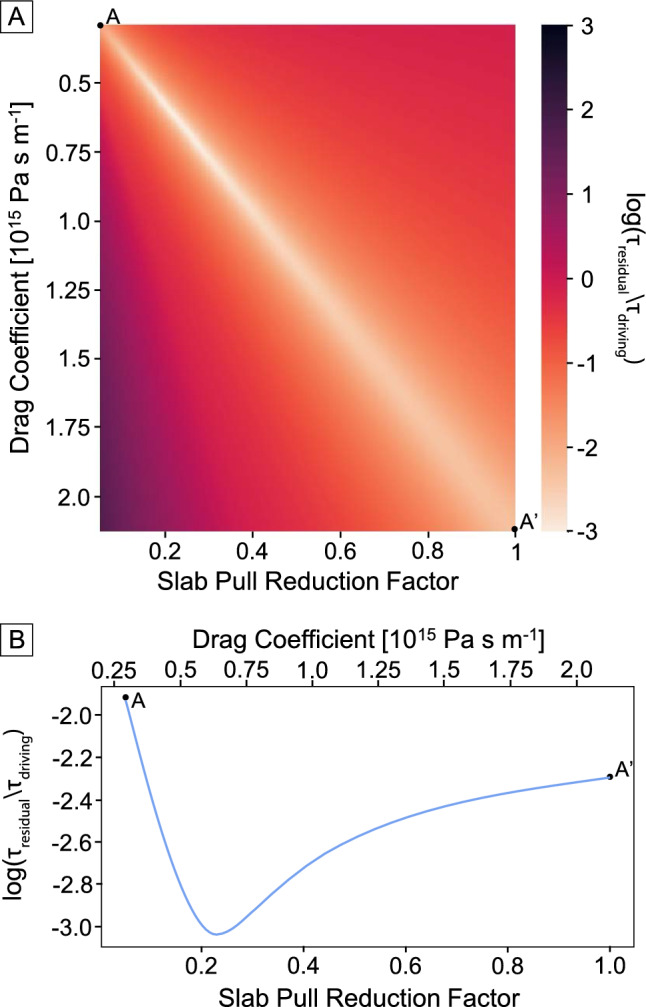
(**A**) Effect of the slab pull reduction factor and drag coefficient on the magnitude of the residual torque at the present-day, averaged for major oceanic plates (Pacific, Nazca and Cocos) for the M2016 model. The residual torque is normalized by the slab pull torque for each plate to ensure that there is no bias towards low slab pull constants in the optimization. For each plate, there is a linear trade off between slab pull reduction factor and drag coefficient. As the largest plate, the Pacific plate dominates the optimization and we highlight the best fit parameters along the diagonal from A to A′ in panel (**B**).

Figure 4 shows the normalized residual torque for each combination of slab pull reduction factor and drag coefficient. The optimum slab pull reduction factor is 0.2–0.25, in agreement with values used in previous studies of plate driving forces^36,43^. The optimum drag coefficient is ~ $6.4 \times 10^{14}$ Pa s m^−1^, which is within the range of drag coefficients used in the recent study of Rowley and Forte^44^ and yields plausible asthenospheric thicknesses and viscosities^63^. This value corresponds to a viscosity of  ~ $1.25 \times 10^{20}$ Pa s for a 200 km thick asthenosphere, which would be consistent with the Haskell constraint of mantle viscosity^64^ for a low viscosity asthenosphere^65^.

In addition to optimizing the drag and slab pull coefficients for the hotspot and geodynamic reference frames, we also analyzed plate driving forces arising from no net rotation (NNR) reference frames applied to each plate model. As NNR frames typically reduce the velocity of the Pacific plate, the total driving force needs to be lower to balance the mantle drag, which leads to lower slab pull reduction factors for a given asthenospheric viscosity and thickness. However, the direction of Pacific plate motion is also slightly more northerly in the NNR frame than in the absolute plate motion reference frames of the models considered. This means that the plate motion is more offset from the westerly GPE force and northwesterly slab pull force, and so the normalized residual is an order of magnitude higher for the NNR reference frame when compared with the M2016 reference frame.

**Figure 5:**
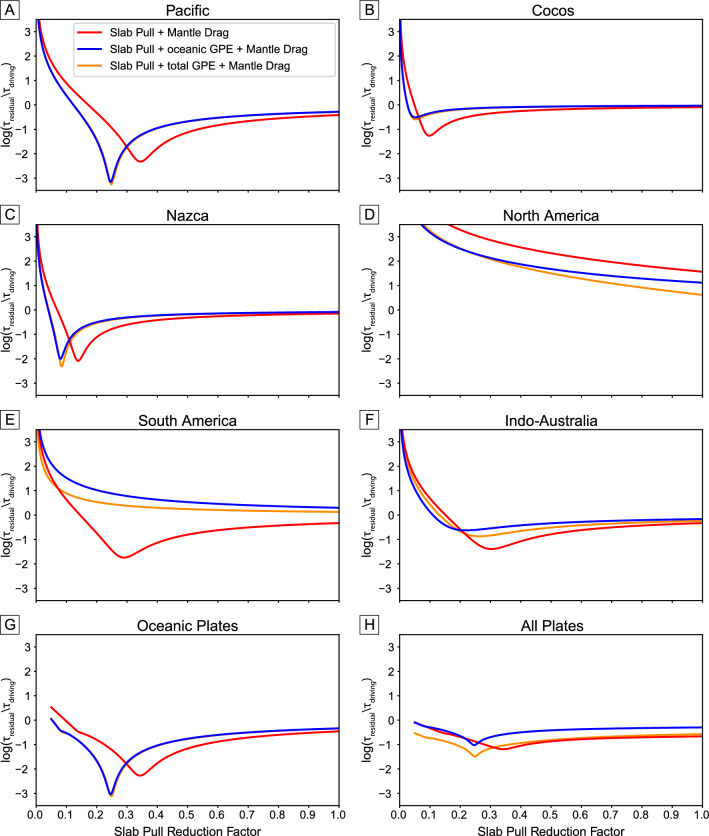
Plots of the residual torque magnitude against slab pull reduction factor, for a selection of present-day plates. The drag coefficient is held constant at $6.4 \times 10^{14} {\text{Pa s m}}^{ - 1}$. The residual torque is normalized by the total driving torque in each scenario and plotted on a log scale, to make identification of the optimum slab pull reduction factor easier. For the oceanic plates (Pacific, Nazca and Cocos) and all plates (excluding those with an area less than 2.5 million km^2^), we plot the area-weighted mean of the log ratios for individual plates.

We also tested the optimization of three different force combinations. Mantle drag always acts as the resisting force, but we varied the driving force to be (i) slab pull alone, (ii) slab pull plus oceanic GPE forces, and (iii) slab pull plus total GPE forces, i.e. including the continental GPE contribution, which we can only easily estimate for the present-day. The general trend shown in Fig. 5 is that continental plates such as North and South America have higher minimum residuals and a broader range of best fit parameters, indicating a poorer fit with plate driving forces compared with the oceanic plates. Another important result is that including GPE forces improves the plate torque balance for the majority of plates as well as the global average. Although we see the best fit when we include the total GPE force, using only oceanic GPE forces produces similar results. The global continental GPE field is poorly constrained in the geological past as very few models include crustal thickness variations through time. This has been recently implemented in M2019 but is still only confined to a few deforming regions, and so we leave implementation of changing continental GPE through time on global scales to future work. Therefore, we only use the oceanic GPE force, derived from seafloor age grids, when computing the plate driving force balance in the geological past, and focus our analysis on oceanic plates.

## Driving forces through time

**Figure 6:**
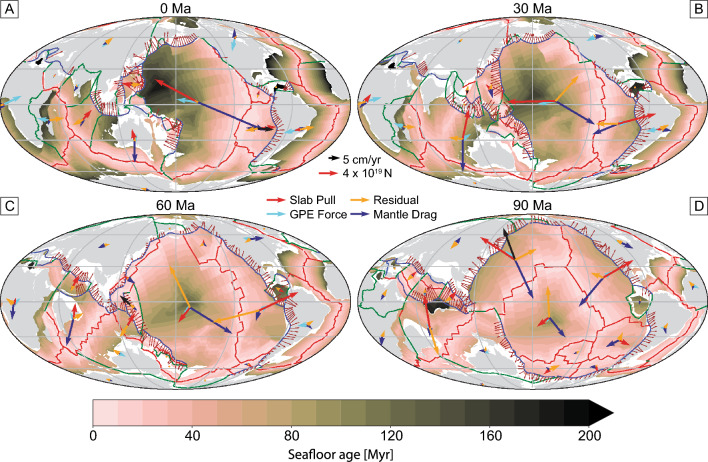
Maps showing slab pull, GPE force, mantle drag and the residual force for each plate at 30 Myr intervals, computed from evaluating the torques at the plate centroids. These maps are showing the M2016 reconstruction model; plots for other reconstruction models can be found in the supplementary material.

Using the optimum parameters and force combinations for the present-day, we calculate plate driving forces back to 120 Ma. Snapshots of the driving forces for each plate are given in Fig. 6 at 30 Myr intervals for the M2016 plate model; for all other reconstructions, see the supplementary information. The vectors show the absolute force acting at the centroid of each plate; for example, the Pacific plate is driven by a slab pull force of $10^{20} {\text{N}}$ and a GPE force of $4 \times 10^{19} {\text{N}}$ at the present day. The slab pull force reduces to $4 \times 10^{19} {\text{N}}$ at 60 Ma, as the total length of subduction zones is lower and the oceanic lithosphere is generally younger, and hence thinner, when entering the subduction zone. The GPE forces acting on the Pacific plate also reduce back in time (to $1.7 \times 10^{19} {\text{N}}$ at 60 Ma), likely due to seafloor age profiles becoming more symmetrical, which leads to tractions cancelling each other out. We also see the residual force increasing back in time; at the present-day, plate driving forces are very well aligned with plate velocities, which yield small residuals after the optimization of parameters described in section “Present-day optimization”. However, the residual becomes a significant component of the force balance at older timesteps.

At 30 Ma, the direction of the slab pull and GPE forces for major plates such as Pacific, Farallon and Indo-Australia are offset with the plate velocity by ~ 30° (Fig. 6B). This leads to residual forces that are on average 60% of the magnitude of the total (slab pull + GPE force) driving force, suggesting that even during Cenozoic times, our parameterization of plate driving forces or understanding of plate kinematic history is incomplete.

**Figure 7:**
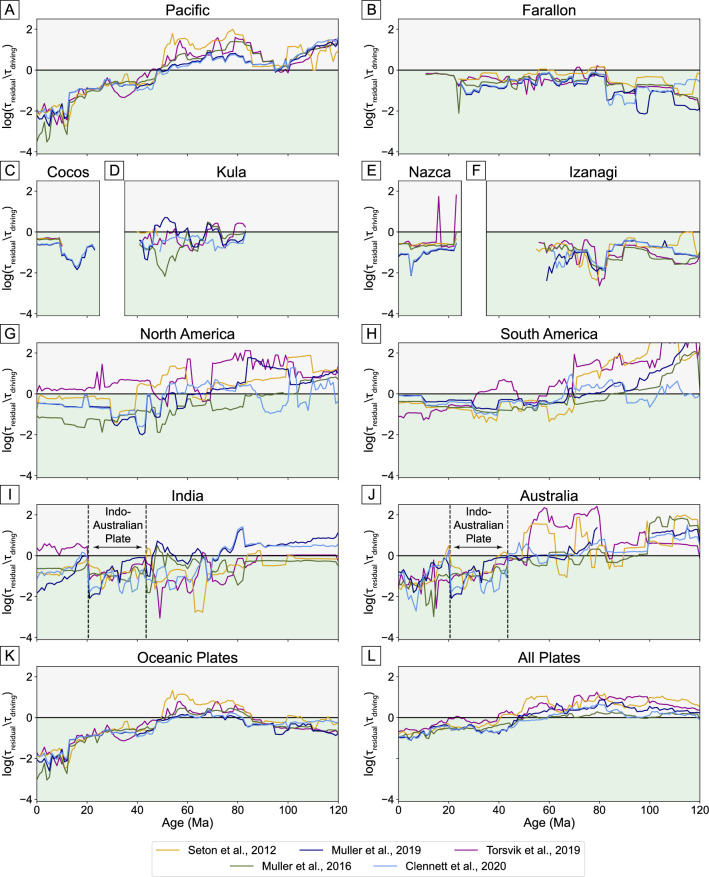
Magnitude of the residual torque, normalized by the driving torque, through time for selected plates and for different plate reconstruction models. Values above zero indicate that the residual torque is greater than the driving torque, which indicates a poor fit between plate motion and driving forces. The slab pull reduction factor and asthenospheric viscosity are optimized for present-day plate motion for each individual model, and then these values are used to calculate the residual of the force balance of slab pull, oceanic GPE and mantle drag and each 1 Myr timestep. For oceanic and all plates, we weight the log ratio for each plate by its area before taking the average.

From 60 to 90 Ma, the residual force for each plate is on average 70% of the magnitude of the total driving force of slab pull and GPE (Fig. 6C,D). This is primarily due to the large residual force for the Pacific plate, which is twice the total driving force. Figure 7A shows a sudden jump in the residual force that occurs at around 47 Ma—the timing of a major plate reorganization event that resulted in the formation of the HEB. The 120° HEB has been attributed either entirely due to a change in the direction of plate motion^66,67^, or southward drift of the Hawaiian hotspot with no change in plate motion^68–70^. Many plate models suggest that the bend was likely caused by a combination of these two end-member scenarios; the models analyzed here show plate motion changes of 20°–40°.

However, the high residual force (Fig. 7A) indicates that current reconstructions of the Pacific plate prior to the HEB are not consistent with our calculated plate driving forces. Of the models analyzed, C2020, which models intra-oceanic subduction in the north Pacific^38–41^, performs best at this time (Fig. S4). However, southwestward subduction of the Pacific plate beneath the Australian plate cancels out this northward force, resulting in a net westward force which cannot explain northward Pacific plate motion. The effects of different subduction zones on Pacific plate motion prior to the HEB are detailed in the “Discussion” section.

The M2016 and T2019 reconstructions also produce a southwest-directed net slab pull force resulting in high residual forces. Although S2012 contains few subduction zones surrounding the Pacific plate at this time, the Pacific Plate moves at 9 cm/yr—faster than the present-day Pacific plate—which results in a large residual force in the direction of plate motion. This is consistent with the conclusions of Faccenna et al.^36^, who suggested that plate motion would have to be driven by active mantle flow at this time. We discuss the potential for the residual force to represent active mantle flow in section “Discussion”.

In addition to the Pacific, other plates also have large jumps in the residual force prior to 50 Ma, suggesting that major plate reorganizations influence the plate driving force balance of other plates in this hemisphere. For example, there was a major reorganization affecting the Australian and Antarctic plates at the same time as the HEB^71^, which could cause a change in driving forces and the jump in residual force seen in Fig. 7J.

The Indian plate shows a more gradual increase in the residual force back in time, but only for M2019 and C2020 (Fig. 7I). These models implement a Neo-tethys ocean plate between the Indian plate and the Neo-tethys subduction zone^72^ which causes a reduced slab pull driving force acting on the Indian plate and thus higher residual forces.

## Discussion

In section “Driving forces through time”, we calculated different plate driving forces for various plate reconstruction models through time, as well as the missing component—or “residual”—required for the forces to sum to zero. Large residuals (on the same order of magnitude or greater than slab pull and GPE force combined) indicate that either plate reconstructions are incorrect in terms of their kinematics and/or that we are missing a significant component of the force balance. This missing contribution could either be a force that we have not considered or parameterized correctly in our simplified analysis, or a missing element of the plate reconstruction model, for example, a missing subduction zone or uncertainties in existing subduction zone geometries. Therefore, we interpret high residuals either as an indication of an unknown, yet strong driving mechanism, such as active mantle flow, or as a proxy for high uncertainty in the plate reconstruction model—the most likely cause being the combination of both. Using the Pacific plate as an example, we now demonstrate how we can identify extra forces and improve the plate reconstruction models.

### Potential influence of mantle flow

For the Pacific plate, the key candidate for an extra force is active mantle flow. Three-dimensional flow is driven by thermal and density variations within the mantle^57,58^. Primarily, this is due to cold, dense slabs sinking beneath current and past subduction zones, which excites mantle flow causing tractions directed towards the downwelling^24–26,34^. A long-lived mantle upwelling, currently centred beneath the East Pacific Rise, has also been suggested to cause asthenospheric flow that can drive the Pacific plate^73^.

Although we did not explicitly calculate active mantle flow, as explained in section “Mantle flow”, we can infer its potential importance as a plate driving force via analysis of the residual. As the mantle density structure represents ~ 200 Myr of subduction, whole mantle flow is generally stable and only changes direction slowly^34^. Based on numerical modelling and scaling relationships, it is estimated that ~ 100 Myr are required to change the large-scale mantle buoyancy structure^74^, indicating that whole mantle flow-generated tractions are not important in rapid plate motion changes. Local force transmission changes due to effects such as slab break off occur on shorter timescales and are, of course, related to convection, but these effects are accounted for in our force balance through the slab pull force calculation.

Smaller-scale effects such as transient plume push^32,33^ might be time-variable on shorter timescales, although still on an order of ~ 10 Myr. However, it is not thought that there were any emplacements of large igneous provinces in the Pacific/Panthallassa region between 90 and 26 Ma^75^, so it is also unlikely that plume push could cause the rapid azimuthal force changes we observe.

**Figure 8:**
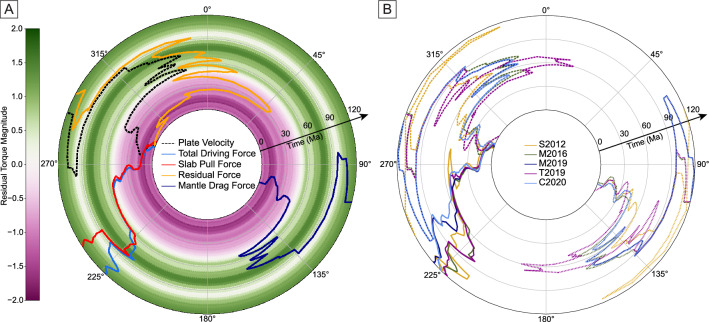
Azimuths of (**A**) plate velocity, total driving force (slab pull and GPE), slab pull, residual force and mantle drag through time for the Pacific plate, according to the M2016 reconstruction model. The colourmap shows the magnitude of the residual force, normalized by the total driving force. (**B**) Total driving force (solid), velocity (dashed) and residual force (dotted) for different reconstructions of the Pacific plate through time. All azimuths are plotted as a 5 Myr running mean, with time increasing in the radial direction. Note that changes in force/velocity azimuth are slightly exaggerated at older times, due to the larger circle size.

Thus, if the residual force from our analysis were to wholly represent mantle flow, it should not change direction on short timescales. To test this, we plotted the azimuths of the different forces, including the residual force, along with the magnitude of the normalized residual force, through time. Figure 8 shows rapid fluctuations in the direction of the residual—due to changes in plate motion—on 10 Myr timescales. While perhaps compatible with some regional effects such as plumes, it is unlikely that a convective mantle flow mechanism would consistently cause such rapid fluctuations. We thus hypothesize that a significant proportion of the Pacific plate’s large residuals could be associated with uncertainties in the plate reconstruction. These rapid azimuthal changes could be a result of the overinterpretation of magnetic anomalies and fracture zones, as the “true” Pacific plate motion could be smoother. Alternatively, the reconstruction could be missing some changes in subduction zone geometry, which would cause an instantaneous change in the slab pull force and plate motion.

### Improving Pacific plate kinematics

If large residual forces are associated with uncertainty in the Pacific plate kinematics from the plate motion models, then an important area to investigate would be the Australia-Pacific plate boundary prior to 45 Ma. This boundary has variously been suggested to be an east-dipping subduction zone^76^, west-dipping subduction zone^77^ or transform margin^36,78,79^. Our plate driving force analysis indicates that the west-dipping subduction implemented in most plate reconstructions (including the five analysed here), which results in a southwestward slab pull on the Pacific plate, is inconsistent with the direction of Pacific plate motion.

**Figure 9:**
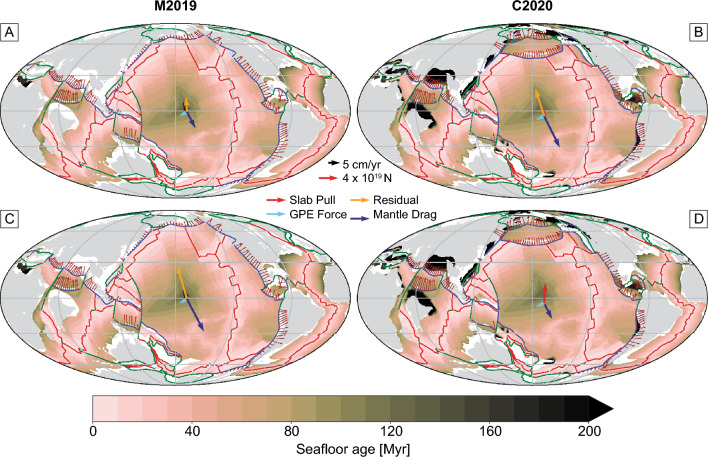
Plate force balance for the pre-HEB Pacific Plate, for modified versions of (**A**,**C**) the M2019 model and (**B**,**D**) the C2020 model, where subduction in the southwest Pacific has been removed. (**A**,**B**) With the present-day optimized parameters of 0.25 and $6.4 \times 10^{14}$ Pa s m^−1^ for slab pull reduction factor and drag coefficient respectively, there is still a large normalized residual torque. (**C**,**D**) Using a slab pull reduction factor of 0.85 and a drag coefficient of $3.2 \times 10^{14}$ Pa s m^−1^, there is still a large residual for the M2019 plate model, which does not include the Kronotsky arc. However, the plate driving forces can be balanced using these parameters for the C2020 reconstruction.

However, as shown in Figs. 6 and 9, implementing either an east-dipping subduction or transform boundary in the southwest Pacific would result in a lack of major subduction of the Pacific Plate, according to S2012, M2016, M2019 and T2019. This scenario still results in major residuals, as there would not be a driving force to balance the large mantle drag force. On the other hand, C2020 implemented a north-dipping intra-oceanic subduction zone in the northern Pacific, which was a simplified scenario based on regional models^38–41^ and seismic tomography constraints^80^. Intra-oceanic subduction beneath the Kronotsky arc has previously been suggested as a driving force for pre-HEB Pacific plate motion^37,40^, and our approach allows us to explicitly test this theory. After removing the southwest Pacific subduction zone, we recomputed the plate driving forces and residual torque at 60 Ma for the C2020 model (Fig. 9). The resulting residual was reduced by 40% compared with the unedited boundaries, and was 20% lower than for models that do not include intra-oceanic subduction in the northern Pacific. This indicates that modelling eastward subduction or transform motion at the Australia-Pacific plate boundary^36,76,78,79^ whilst including northward subduction of the Pacific beneath the Kronotsky arc can slightly improve the fit with plate motion. However, the residual torque is still an order of magnitude greater than the driving torque due to the large drag force; the torques can only be balanced by increasing the slab pull reduction factor to 0.85 and halving the asthenospheric viscosity to $6.4 \times 10^{19}$ Pa s, for a 200 km thick asthenosphere. Since there is no evidence that these parameters would significantly change at 60 Ma, this implies that slab suction and other mantle flow forces were potentially more important in controlling the magnitude of plate velocity in the past^35^. However, the azimuthal match between slab pull and plate motion is significantly improved. This is in agreement with the work by Hu et al.^37^, who found that mantle density-driven plate motions better matched modelled Pacific plate motion when intra-oceanic subduction was included in the reconstruction. Using global mantle flow models, they showed that cessation of intra-oceanic subduction in the northern Pacific caused a 30°–35° change in plate motion, with the other 25°–30° of the HEB being attributed to hotspot drift.

In summary, we suggest that modifying reconstructions of the southwest and northern Pacific from ~ 50 to 90 Ma can improve the balance of plate driving forces. We substantiate that active mantle flow is important in driving the Pacific plate prior to the HEB, but is unlikely to explain rapid fluctuations in the azimuth of Pacific plate motion, which are likely due to remaining uncertainties in plate reconstruction models.

### Optimizing geodynamics in plate reconstructions

Another source of uncertainty in a plate reconstruction comes from the choice reference frame applied to constrain absolute plate motions. Tetley et al.^20^ attempted to address these uncertainties by optimizing global reference frames, minimizing the misfit of plate models to observed hotspot tracks and applying prior geodynamic assumptions that net rotation should be small but non-zero and subduction trenches should mainly retreat with small velocities; this optimized reference frame is included in the M2019 and C2020 models. Although the reference frame optimization does not explicitly consider plate driving forces, the two models that include this reference frame have the lowest residual forces over the 50–90 Ma period where plate driving forces are most poorly balanced (Fig. 7). These models also have lower residuals than their corresponding no net rotation reference frames. Although net rotation is inferred to be small for the present-day^18,19^, minimizing net rotation alone (i.e. no net rotation reference frames) without considering subduction zone kinematics or hotspot tracks does not provide the best fit with respect to plate driving forces.

### Towards plate driving force-constrained plate reconstructions

In section “Improving Pacific plate kinematics”, we presented an example of how constraints from plate driving forces can be used to improve plate reconstruction models. This can provide a template for producing more geodynamically-plausible plate reconstructions in the future. Once researchers have constructed their plate models based on geological or geophysical observations, they could efficiently run our algorithm to calculate the plate driving force balance. The steps are to (1) load in the plate polygons and rotation files and seafloor age grids; (2) run the compute forces code and save the outputs; (3) run the optimization code for certain plates at the present-day, or instead use our best-fit parameters for slab pull reduction factor and asthenospheric viscosity; (4) make map plots as well as plots of the residual through time to find areas with large residual forces. Optional extra steps include adding forces or changing parameters within our algorithm, which could potentially improve the force balance. Such additions could also form the basis of future studies investigating different mechanisms that drive plate motion. We provide a *Jupyter* notebook with detailed comments to help users to run these steps. Any resulting large residual forces could indicate potential uncertainty and point towards regions and time periods that need investigating in more detail.

## Conclusions

We present a method to calculate plate driving forces, including slab pull, lithospheric thickening, mantle drag resistance and the total residual (or missing) component for any plate reconstruction and time of interest. Substantiating earlier work, we find that slab pull reduction factors of 0.2–0.25, for 700 km long slabs, and asthenospheric viscosities of $\sim 1.25 \times 10^{20}$ Pa s, for a 200 km thick asthenosphere, lead to good plate motion fits for the present-day. However, there is a poorer fit between plate motion and plate driving forces in the geological past; in all models, the residual force makes up almost 40% of the driving force at times as recent as 30 Ma, and this increases to over 50% of the driving force between 90 and 50 Ma.

Large residual components indicate that there is high uncertainty in either the plate reconstruction model or in the forces that we assume in our calculation. We choose the Pacific Plate at around 60 Ma as an example of how we can improve a plate reconstruction models, with our analysis indicating that modifying the southwest and northern Pacific boundaries can improve the model with respect to the plate driving force balance. Through analysis of the residual force, we infer that active mantle flow is unlikely to have caused the rapid changes in the azimuth of Pacific plate motion, which we suggest are more likely due to uncertainties in the plate model. Our method can be used by the plate modelling community to evaluate whether plate reconstructions produce a good fit with a given set of plate driving forces, and thus form part of a workflow leading towards dynamically consistent plate reconstructions.

## Supplementary Information

Supplementary Information.

## Data Availability

The code used to do the analysis in this paper can be downloaded at: 10.5281/zenodo.7904975. This code also includes scripts to recreate the figures, which were created in matplotlib^81^ and cartopy^82^ using scientific colour maps^83^. The S2012, M2016, M2019 and C2020 models can be downloaded from https://www.earthbyte.org/category/resources/data-models/global-regional-plate-motion-models/ and T2019 can be found at: http://www.earthdynamics.org/earthmodel/page6.html.
