# Supplementary material for: Assessing plate reconstruction models using plate driving force consistency tests
Source: Sci Rep. 2023 Jun 23;13:10191. doi: 10.1038/s41598-023-37117-w (PMC10290141; doi:10.1038/s41598-023-37117-w)
Supplement: Supplementary file 1 — Supplementary Information. [file 41598_2023_37117_MOESM1_ESM.pdf]

Supplement for "Assessing plate reconstruction models using plate driving force consistency tests"

Edward J. Clennett[1,2], Adam F. Holt[3], Michael G. Tetley[2], Thorsten W. Becker[1,2,4] and Claudio Faccenna[5,6]

[1]Department of Geological Sciences, Jackson School of Geosciences, The University of Texas at Austin, USA.
[2]Institute for Geophysics, Jackson School of Geosciences, The University of Texas at Austin, USA.
[3]Rosenstiel School of Marine, Atmospheric, and Earth Science, University of Miami, USA.
[4]Oden Institute for Computational Engineering & Sciences, The University of Texas at Austin, USA.
[5]Dipartimento Scienze, Università Roma Tre, Italy.
[6]GFZ Helmholtz Centre Potsdam, German Research Centre for Geosciences, Germany.

Contents of this file
Table S1
Figures S1-S6

Parameter	Value
Reference mantle density, $\boldsymbol{\rho_m}$	$3300\ \mathrm{kg\ m^{-3}}$
Thermal expansivity, $\boldsymbol{\alpha}$	$3 \times 10^{-5}\ \mathrm{K^{-1}}$
Thermal diffusivity, $\boldsymbol{\kappa}$	$10^{-6}\ \mathrm{m^2 s^{-1}}$
Mantle-slab temperature difference, $\boldsymbol{\Delta T}$	$1200\ \mathrm{K}$
Acceleration due to gravity, $\boldsymbol{g}$	$9.81\ \mathrm{m\ s^{-2}}$
Density of seawater, $\boldsymbol{\rho_w}$	$1020\ \mathrm{kg\ m^{-3}}$
Density of oceanic crust, $\boldsymbol{\rho_{oc}}$	$2868\ \mathrm{kg\ m^{-3}}$
Lithospheric density, $\boldsymbol{\rho_l}$	$3412\ \mathrm{kg\ m^{-3}}$
Asthenospheric density, $\boldsymbol{\rho_a}$	$3350\ \mathrm{kg\ m^{-3}}$
Density of continental crust, $\boldsymbol{\rho_{cc}}$	$2861\ \mathrm{kg\ m^{-3}}$
Density of continental mantle, $\boldsymbol{\rho_{cm}}$	$3380\ \mathrm{kg\ m^{-3}}$
Compensation depth, $\boldsymbol{L}$	$130\ \mathrm{km}$
Lithospheric shell thickness, $\boldsymbol{L_0}$	$100\ \mathrm{km}$

Table S1. Values of parameters used in this study, after Turcotte and Schubert (1982) and Becker and O'Connell (2001).

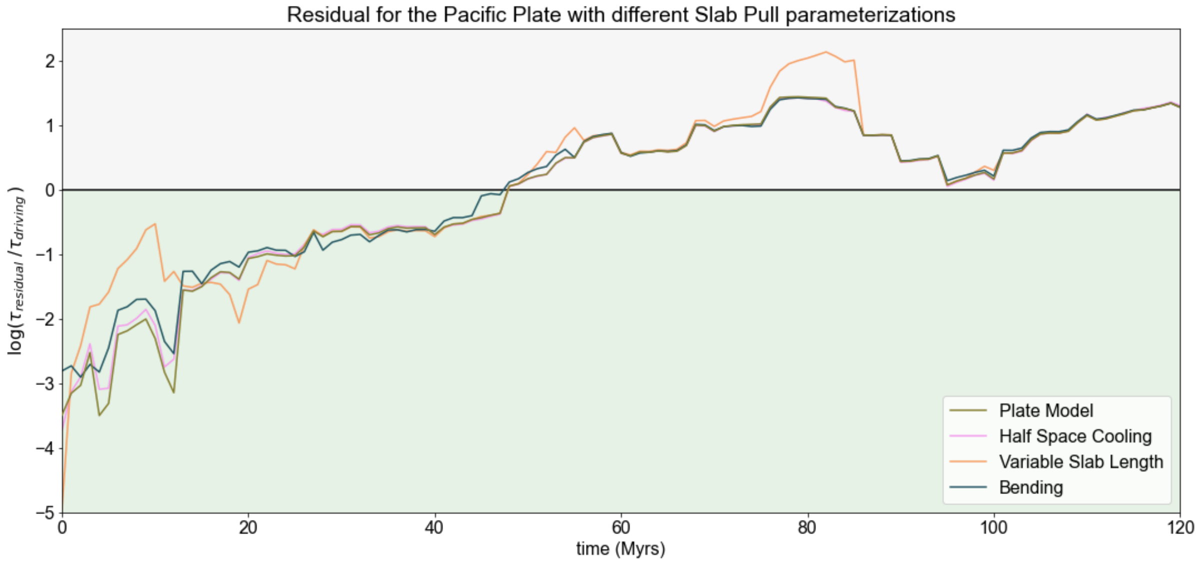

Figure S1. Magnitude of the residual torque, normalized by the driving torque of slab pull and GPE tractions, for different slab pull parameterizations. The different ways of calculating slab pull are detailed in section 2.1 of the main text.

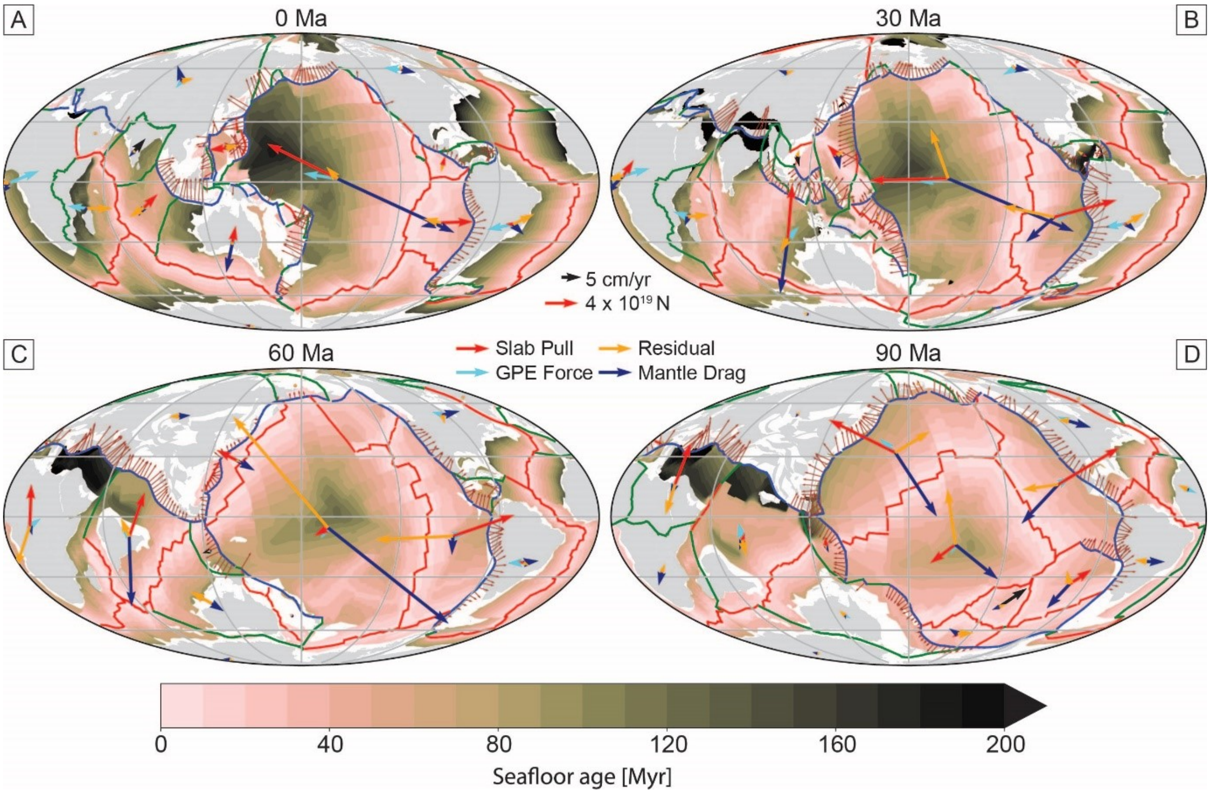

Figure S2. Maps showing slab pull, GPE force, mantle drag and the residual force at 30 Myr intervals, from the Seton et al. (2012) reconstruction model.

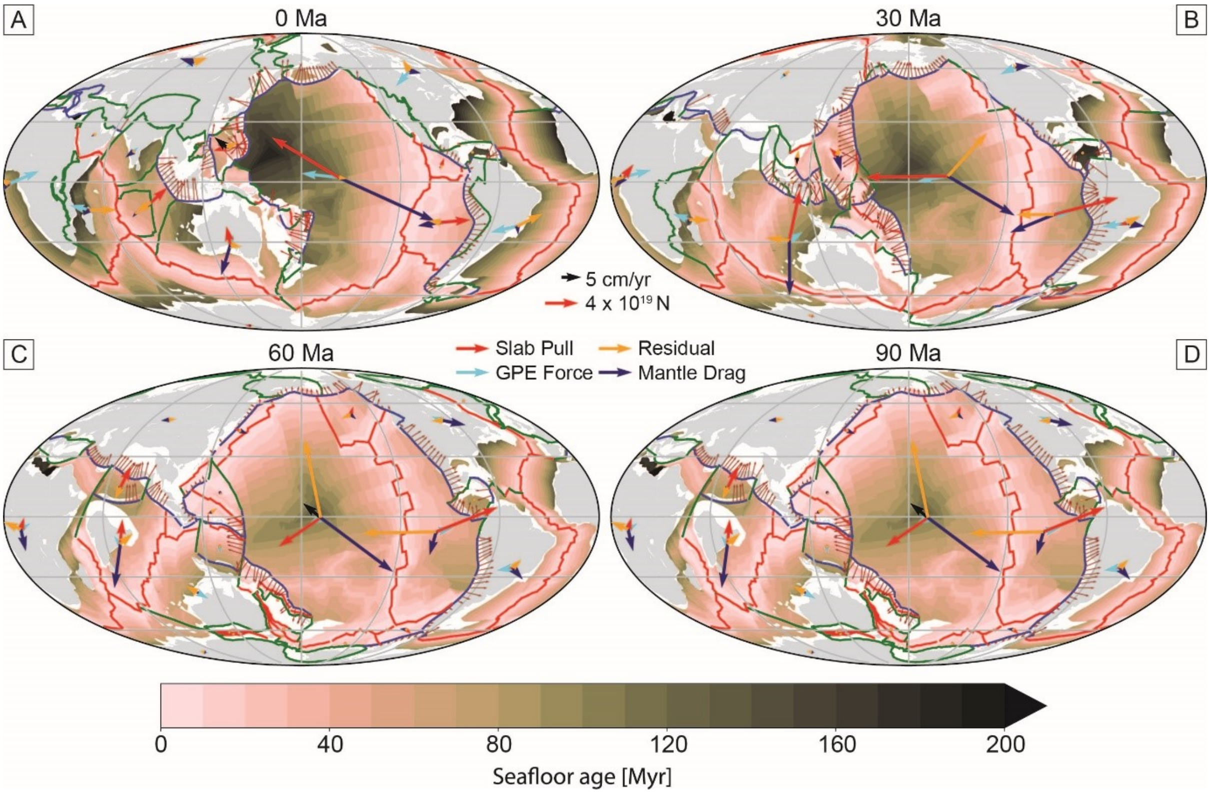

Figure S3. Maps showing slab pull, GPE force, mantle drag and the residual force at 30 Myr intervals, from the Müller et al. (2019) reconstruction model.

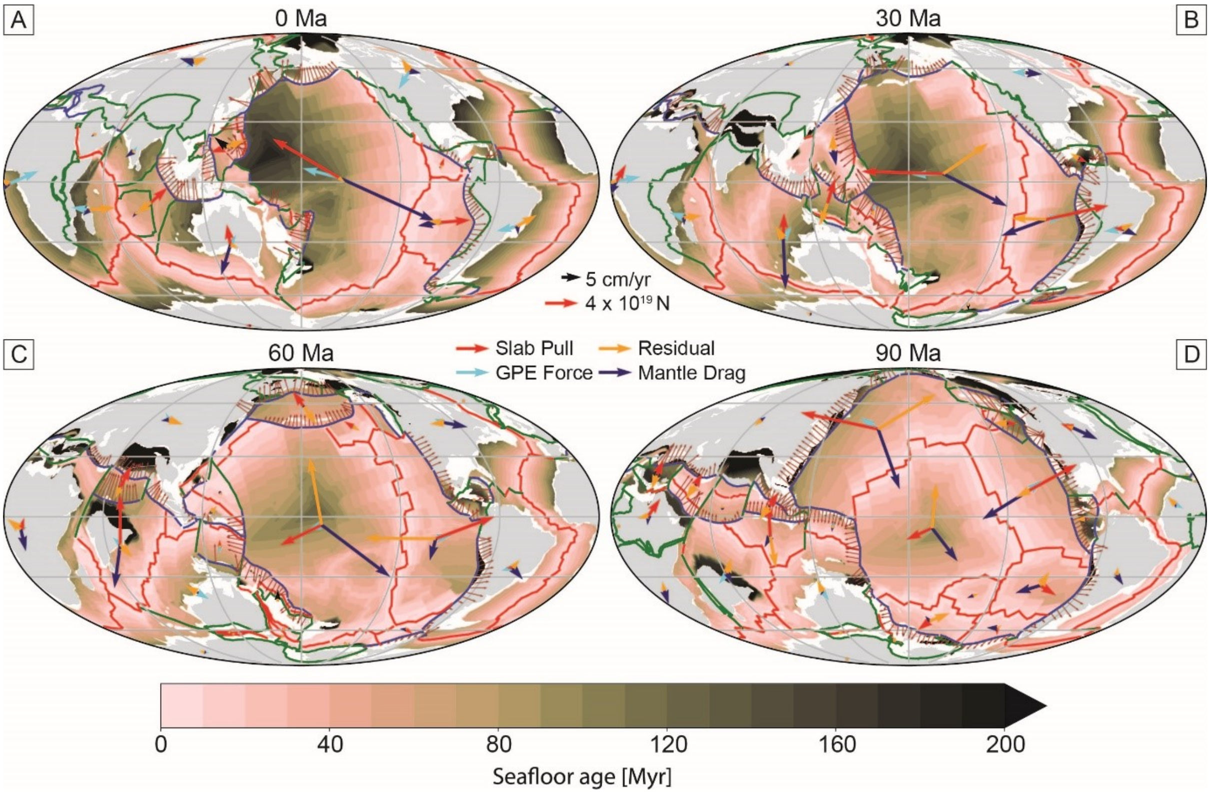

Figure S4. Maps showing slab pull, GPE force, mantle drag and the residual force at 30 Myr intervals, from the Clennett et al. (2020) reconstruction model.

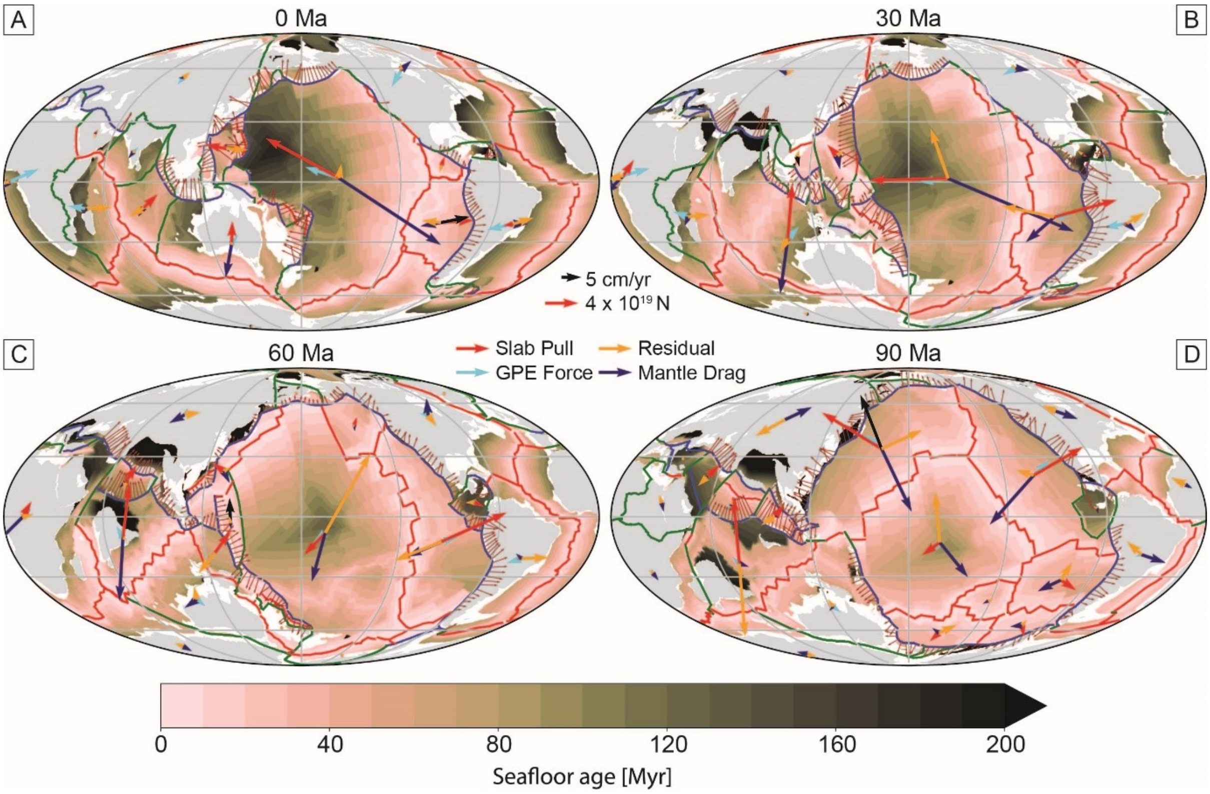

Figure S5. Maps showing slab pull, GPE force, mantle drag and the residual force at 30 Myr intervals, from the Torsvik et al. (2019) reconstruction model.

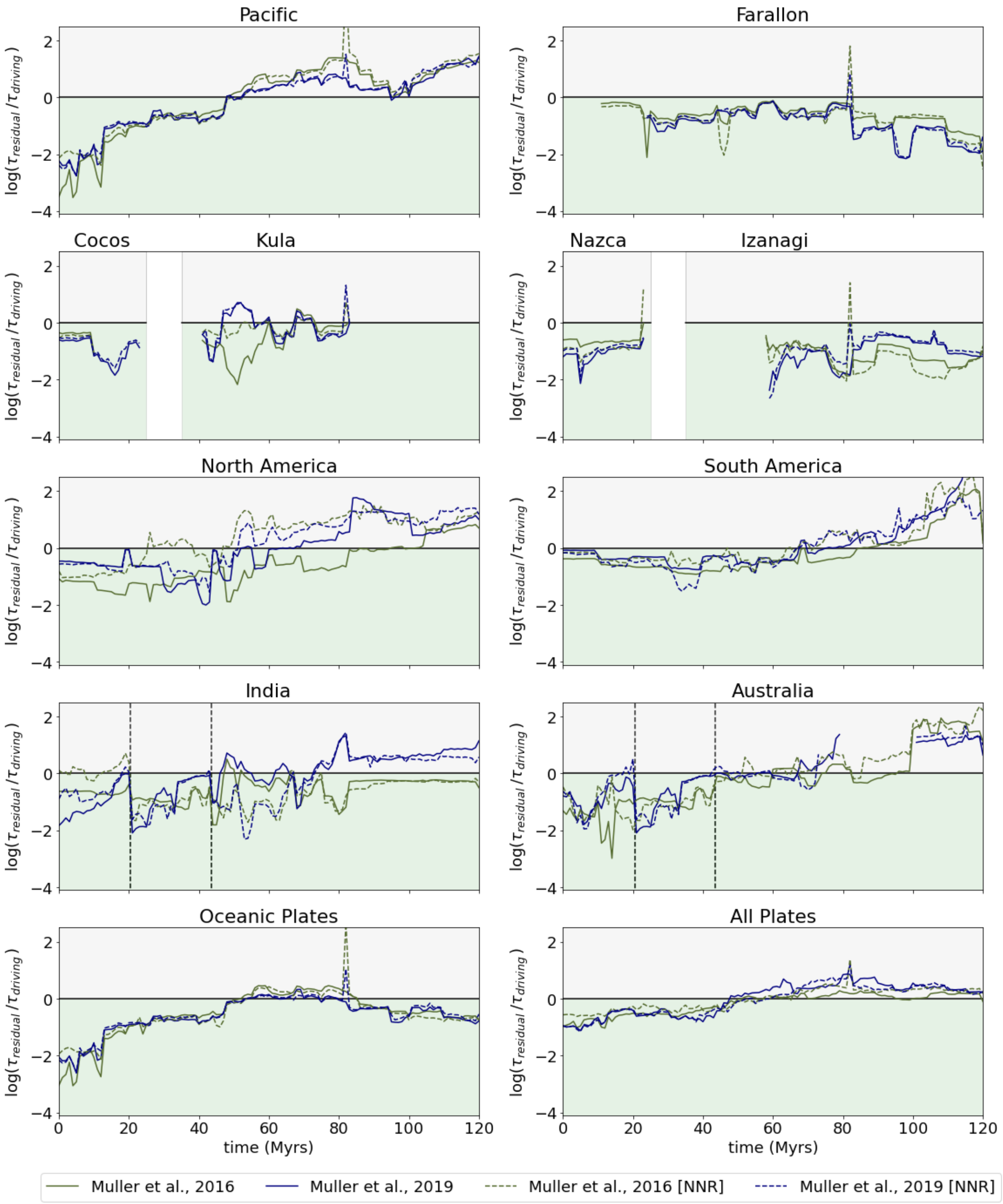

Figure S6. Magnitude of the residual torque, normalized by the driving torque of slab pull and GPE tractions, for the Müller et al. (2016) and Müller et al. (2019) plate reconstruction models for both the standard hybrid reference frame of each model (solid line), and a no net rotation (NNR) reference frame. For oceanic and all plates, the area-weighted average residual is plotted.

References

Becker, T. W., & O'Connell, R. J. (2001). Predicting plate velocities with mantle circulation models. *Geochemistry, Geophysics, Geosystems*, *2*(12), 2001GC000171. https://doi.org/10.1029/2001GC000171

Clennett, E. J., Sigloch, K., Mihalynuk, M. G., Seton, M., Henderson, M. A., Hosseini, K., Mohammadzaheri, A., Johnston, S. T., & Müller, R. D. (2020). A Quantitative Tomotectonic Plate Reconstruction of Western North America and the Eastern Pacific Basin. *Geochemistry, Geophysics, Geosystems*, *21*(8), e2020GC009117. https://doi.org/10.1029/2020gc009117

Müller, R. D., Zahirovic, S., Williams, S. E., Cannon, J., Seton, M., Bower, D. J., Tetley, M. G., Heine, C., Le Breton, E., Liu, S., Russell, S. H. J., Yang, T., Leonard, J., & Gurnis, M. (2019). A Global Plate Model Including Lithospheric Deformation Along Major Rifts and Orogens Since the Triassic. *Tectonics*, *38*(6), 1884–1907. https://doi.org/10.1029/2018TC005462

Seton, M., Müller, R. D., Zahirovic, S., Gaina, C., Torsvik, T., Shephard, G., Talsma, A., Gurnis, M., Turner, M., Maus, S., & Chandler, M. (2012). Global continental and ocean basin reconstructions since 200Ma. *Earth-Science Reviews*, *113*(3–4), 212–270. https://doi.org/10.1016/j.earscirev.2012.03.002

Torsvik, T. H., Steinberger, B., Shephard, G. E., Doubrovine, P. V., Gaina, C., Domeier, M., Conrad, C. P., & Sager, W. W. (2019). Pacific-Panthalassic Reconstructions: Overview, Errata and the Way Forward. *Geochemistry, Geophysics, Geosystems*, *20*(7), 3659–3689. https://doi.org/10.1029/2019GC008402

Turcotte, D. L., & Schubert, G. (1982). *Geodynamics: Applications of Continuum Physics to Geological Problems* (1st ed.). John Wiley.
